# Urgent request for pretomanid label expansion to align with WHO guidelines and improve treatment accessibility and efficacy

**DOI:** 10.5588/ijtldopen.25.0152

**Published:** 2025-03-12

**Authors:** L. Kuksa, C. Andrejak, B. Haecker, G. Bothamley, A. Calcagno, D.M. Cirillo, R. Duarte, R. Fatima, G. Ferlazzo, L. Guglielmetti, G. Günther, C. Hewison, C.R. Horsburgh, T. Jäger, Y. Kalancha, R. Otto-Knapp, K. Kranzer, T. Lillebaek, G. Marks, K. Middelkoop, I. Motta, V. Rabinova, P. Sommerfeld, P. Tattevin, C. Lange

**Affiliations:** ^1^ Riga East University Hospital, Tuberculosis and Lung Disease center, Riga, Latvia;; ^2^ Pneumology Department, University Hospital Centre Amiens-Picardie, Amiens, France;; ^3^ German Central Committee against Tuberculosis (DZK), Berlin, Germany;; ^4^ Homerton University Hospital, London, UK;; ^5^ London School of Hygiene & Tropical Medicine, London, UK;; ^6^ Unit of Infectious Diseases, Department of Medical Sciences, University of Torino, Torino, Italy;; ^7^ IRCCS San Raffaele Scientific Institute, Milan, Italy;; ^8^ EPIUnit ITR, Instituto de Saúde Pública da Universidade do Porto, Porto, Portugal;; ^9^ Estudos das Populações, ICBAS - Instituto de Ciências Biomédicas Abel Salazar, Universidade do Porto, Porto, Portugal;; ^10^ INSA – Instituto de Saúde Pública Doutor Ricardo Jorge, INSA Porto, Porto, Portugal;; ^11^ United Nations Office for Project Services (UNOPS), Islamabad, Pakistan;; ^12^ Médecins Sans Frontières Access Campaign, Geneva, Switzerland;; ^13^ Sorbonne Université, Institut national de la santé et de la recherche médicale, Unité 1135, Centre d'Immunologie et des Maladies Infectieuses, Paris France;; ^14^ Assistance Publique-Hôpitaux de Paris, Hôpital Pitié-Salpêtrière, Centre National de Référence des Mycobactéries, Paris, France;; ^15^ Department of Pulmonology, Allergology and Clinical Immunology, Inselspital, Bern University Hospital, University of Bern, Bern, Switzerland;; ^16^ Médecins Sans Frontières, Tuberculosis, Working Group, Paris, France;; ^17^ Boston University School of Public Health, Department of Global Health, Boston, MA, USA;; ^18^ German Center for Infection Research (DZIF), Braunschweig, Germany;; ^19^ TB Europe Coalition, Kyiv, Ukraine;; ^20^ Clinical Research Department, London School of Hygiene & Tropical Medicine, UK;; ^21^ The Health Research Unit Zimbabwe, Biomedical Research and Training Institute, Zimbabwe;; ^22^ Division of Infectious Diseases and Tropical Medicine, University Hospital, LMU Munich, Munich, Germany;; ^23^ German Center for Infection Research (DZIF), partner site Munich, Germany;; ^24^ International Reference Laboratory of Mycobacteriology, Statens Serum Institut, Copenhagen, Denmark;; ^25^ Global Health Section, Department of Public Health, University of Copenhagen, Copenhagen, Denmark;; ^26^ The Woolcock Institute, of Medical Research, Macquarie Park, NSW, Australia;; ^27^ Division of Global Health, Burnet Institute, Melbourne, VIC, Australia;; ^28^ Institute of Infectious Disease and Molecular Medicine, University of Cape Town, Cape Town;; ^29^ Desmond Tutu HIV Centre, Department of Medicine, University of Cape Town, Cape Town;; ^30^ MRC Clinical Trials Unit, University College London, London, UK;; ^31^ TB Alert, London, UK;; ^32^ Infectious Diseases & Intensive Care Unit, Pontchaillou University Hospital, Rennes, France;; ^33^ Department of Clinical Infectious Diseases, Research Center Borstel, Leibniz Lung Center, Borstel, Germany;; ^34^ German Center for Infection Research (DZIF), Partner Site Hamburg-Lübeck-Borstel-Riems, Germany;; ^35^ Respiratory Medicine & International Health, University of Lübeck, Lübeck, Germany;; ^36^ Baylor College of Medicine and Texas Children's Hospital, Global Tuberculosis Program, Houston, TX, USA.

**Keywords:** tuberculosis, drug resistance, MDR-TB, RR-TB, XDR-TB, European Medicines Agency

## Abstract

Pretomanid is a key anti-TB drug included in the WHO list of essential medications. The current EMA-approved label for pretomanid restricts its use to the regimen comprising bedaquiline, pretomanid and linezolid (BPaL) and only for extensively drug-resistant-TB or multidrug-resistant TB, “when antibiotics used for the latter form of TB do not work or cause unacceptable side effects.” This restricted use implies that the older, prolonged and poorly tolerated regimens remain the recommended treatment for most cases of drug-resistant TB. The authors, representing many respiratory groups and societies, call for the label expansion of pretomanid to align with global guidelines, allowing for broader use.

We express our strong support for the proposed label expansion of pretomanid, a key anti-TB drug, which is included on the WHO model list of essential medicines.^1^ This initiative aims to align clinical practice with the latest WHO guidelines^2^ and updated guidelines by the American Thoracic Society, U.S. Centers for Disease Control and Prevention, European Respiratory Society, and Infectious Diseases Society of America.^3^ The current EMA-approved label for pretomanid (Dovprela^®^) tablets restricts its use to the regimen comprising bedaquiline, pretomanid and linezolid (BPaL) and only for extensively drug-resistant-TB (XDR-TB) or for multidrug-resistant (MDR-TB) “when antibiotics used for this form of tuberculosis do not work or cause unacceptable side effects”.^4^ In this context, it is important to highlight that the definition of XDR-TB was revised by WHO in 2021, after pretomanid (Dovprela^®^) authorisation, and now describes a different patient population,^5^ those with *Mycobacterium tuberculosis* resistance against rifampicin (with or without isoniazid), levofloxacin or moxifloxacin, and bedaquiline and/or linezolid.

This restricted use implies that the older, prolonged and poorly tolerated individualised regimens remain the recommended treatment for most persons affected by MDR-TB or rifampicin-resistant TB (RR-TB). However, since 2022, and based on evidence from the NiX, ZeNix and TB-PRACTECAL trials, WHO has recommended BPaL with moxifloxacin (BPaLM) as the standard of care for MDR/RR-TB. Furthermore, WHO advises omitting moxifloxacin in cases of fluoroquinolone resistance.^6–8^ Following the WHO 2022 guidelines recommending programmatic use of BPaL(M) for all patients with rifampicin-resistant TB, WHO issued a Call to Action^9^ emphasising that rapid implementation of these regimens could significantly improve treatment outcomes and patient quality of life. WHO urged governments, healthcare providers and stakeholders to prioritise the integration of BPaL(M) into national TB programs.

The BPaL(M) regimen has been implemented in individual countries through pilot programs, clinical trials, operational research and programmatic settings. Notably, the mBPaL trial in India,^10^ a prospective cohort study in Belarus and Uzbekistan,^11^ and a pilot implementation study in Pakistan,^12^ demonstrated high success rates and a safety profile consistent with the results of the earlier Nix-TB, ZeNix and TB-PRACTECAL trials. Furthermore, the BPaL(M) regimen has been shown to be cost-effective across four countries.^13,14^ Shortening the duration of treatment is a priority for MDR/RR-TB patients, along with reducing pill burden and tolerability of new regimens.^15^ However, recent data from TBNet and WHO-EURO surveys assessing the availability of drugs and resistance testing in Europe highlight significant disparities in access to the BPaL(M) regimen. These findings reveal that of the surveyed countries, only 23/44 (52%) and 3/18 (17%), respectively, have full access to all necessary drugs, with pretomanid being notably the least accessible.^7,16^

In EU/EEA countries, numbers of MDR/RR-TB cases are increasing. Therefore, we urge an expedited review of the pretomanid (Dovprela®) label by the EMA. A recent review supports the use of pretomanid-based regimens in patients with drug-resistant TB and found that pretomanid-based regimens are efficacious with tolerable safety profile.^17^ An updated label that includes broader indications for pretomanid will not only align with the best clinical practices and WHO recommendations but also address the urgent need for equitable access to treatment across Europe.

We strongly advocate for a label update that reflects the WHO’s current guidelines on use of pretomanid to improve treatment accessibility and efficacy.
